# Anaemia Among School-Going Adolescent Girls in India: A Systematic Review of Prevalence, Predictors, and Prevention Pathways

**DOI:** 10.7759/cureus.104980

**Published:** 2026-03-10

**Authors:** Sagarika Duggirala, Saptarishi Bose, Revathi Devi Karuturi

**Affiliations:** 1 Community Medicine, Great Eastern Medical School and Hospital, Srikakulam, IND; 2 Child Health, Sudhir Heart Centre, Berhampur, IND

**Keywords:** adolescent girls, anaemia, india, iron deficiency, public health, school health

## Abstract

Anaemia remains a major public health challenge among adolescent girls in India, with persistently high prevalence despite long-standing national nutrition programmes. School-going adolescent girls represent a critical subgroup due to increased physiological iron requirements during adolescence and the long-term consequences for growth, cognition, and future maternal health. This systematic review, conducted in accordance with Preferred Reporting Items for Systematic Reviews and Meta-Analyses (PRISMA) 2020 guidelines, synthesised evidence from studies published between January 2010 and December 2024 across major databases and grey literature sources. Observational and interventional studies reporting anaemia prevalence, predictors, or preventive strategies among school-going adolescent girls (10-19 years) in India were included. Eleven studies comprising national surveys and regional school-based investigations were analysed. Reported anaemia prevalence ranged from approximately 40% to over 70%, with consistently higher burden in socioeconomically disadvantaged and high-risk regions. Key determinants included inadequate dietary iron intake, menstrual blood loss, poor sanitation, helminthic infections, and suboptimal adherence to iron-folic acid supplementation programmes. Evidence suggests that integrated school-based interventions combining supplementation, nutrition education, and screening are more effective than single-component strategies. Overall, anaemia among school-going adolescent girls in India remains a persistent and inequitable public health problem, requiring strengthened implementation of context-specific, school-based prevention strategies and improved programme monitoring.

## Introduction and background

Adolescence is a critical period of rapid physical, psychological, and cognitive development, during which nutritional requirements increase substantially [1]. Anaemia remains highly prevalent globally and continues to disproportionately affect adolescent girls in low- and middle-income countries [2]. For adolescent girls, the onset of menarche and accelerated growth further elevate iron requirements, increasing vulnerability to iron deficiency and anaemia [3]. In India, adolescent girls constitute a large demographic group but continue to experience a high burden of anaemia due to inadequate dietary intake, gender-based nutritional inequities, and socioeconomic disadvantage [4].

Anaemia during adolescence has far-reaching consequences, including impaired physical growth, reduced cognitive function, poor academic performance, and increased susceptibility to infections [5]. Moreover, anaemia in adolescence adversely affects future reproductive outcomes, contributing to maternal morbidity, low birth weight, and the perpetuation of intergenerational malnutrition [6]. Despite national initiatives such as the Weekly Iron and Folic Acid Supplementation programme and Anaemia Mukt Bharat, anaemia prevalence among adolescent girls has declined only marginally [7,8].

While previous reviews have examined anaemia among adolescents or women of reproductive age, focused synthesis of evidence specific to school-going adolescent girls, particularly emphasising prevention pathways and implementation gaps, remains limited. This systematic review addresses this gap by synthesising evidence on the burden, determinants, and preventive strategies for anaemia among school-going adolescent girls in India.

## Review

Methods

Study Design and Reporting

This systematic review was conducted in accordance with the Preferred Reporting Items for Systematic Reviews and Meta-Analyses (PRISMA) 2020 guidelines [9].

Search Strategy

A comprehensive literature search was conducted in PubMed, Scopus, Web of Science, Google Scholar, and the Cochrane Library for studies published between January 2010 and December 2024. Search terms combined anaemia-related keywords with adolescent, school-going status, and India-specific terms. Grey literature sources, including the World Health Organization (WHO), United Nations Children’s Fund (UNICEF), and Ministry of Health and Family Welfare portals, were also screened. An example PubMed search string is provided to enhance reproducibility: ("anaemia" OR "anemia" OR "iron deficiency") AND ("adolescent girls" OR "school-going adolescents") AND ("India") AND ("prevalence" OR "risk factors").

Electronic Search Strategy

The PubMed search strategy included combinations of anaemia-related terms, population descriptors, and geographic identifiers. Filters applied were publication date January 2010 to December 2024, English language, and human studies. Additional databases (Scopus, Web of Science, Google Scholar, and the Cochrane Library) were searched using equivalent keyword combinations. For example, the Scopus search strategy included ("anaemia" OR "anemia" OR "iron deficiency") AND ("adolescent girls" OR "school-going adolescents") AND ("India") AND ("prevalence" OR "risk factors"). Detailed search strategies used for each database are provided in the Appendices.

Eligibility Criteria

Studies conducted in India among school-going adolescent girls aged 10-19 years and reporting anaemia prevalence, predictors, or preventive interventions were included. Reviews, editorials, case reports, and studies without primary anaemia data were excluded. Foundational pre-2010 studies with national coverage or strong methodological relevance were retained to provide historical baseline comparisons and assess temporal trends in anaemia prevalence. Strong methodological relevance was defined as studies with nationally representative samples, robust sampling methodology, or use of standardised haemoglobin assessment techniques. Where reported, anaemia definitions were based on study-specific criteria, most commonly aligned with World Health Organization haemoglobin cut-offs; however, variability in thresholds across studies was acknowledged and considered during interpretation.

Study Selection

Two reviewers independently screened titles and abstracts for eligibility, followed by full-text assessment of potentially relevant articles. Discrepancies were resolved through discussion and consensus, with consultation of a third reviewer when necessary.

The database search yielded 1,160 records. After removal of records prior to screening, 892 records were screened. A total of 144 full-text articles were assessed for eligibility, of which 11 studies met the inclusion criteria and were included in the final qualitative synthesis.

Data Extraction

Data were extracted independently by two reviewers using a standardised data extraction form capturing study characteristics, population details, haemoglobin assessment methods, anaemia prevalence, and reported predictors and prevention strategies. Any disagreements were resolved by consensus. A third reviewer contributed to data interpretation, manuscript drafting, and critical revision for important intellectual content. Where reported, consideration of confounding factors (e.g., socioeconomic status, dietary patterns, and menstrual factors) within individual studies was noted during data extraction and considered in the interpretation of findings. Prevalence estimates were extracted as reported in the original studies, with no additional weighting or recalculation performed due to methodological heterogeneity across survey and school-based studies.

Quality Assessment

Methodological quality was assessed using Joanna Briggs Institute (JBI) critical appraisal tools, and studies were categorised as low, moderate, or high quality [10]. Study quality informed interpretation of findings, with greater emphasis placed on national surveys and studies rated as high quality. A summary quality appraisal table presents methodological ratings of all included studies using the Joanna Briggs Institute criteria. Quality appraisal was performed independently by two reviewers, with disagreements resolved through discussion and consensus. Formal calibration exercises were not conducted, but predefined JBI criteria were applied consistently.

Data Synthesis

Owing to substantial heterogeneity in study designs, population characteristics, haemoglobin assessment methods, anaemia definitions, and outcome reporting, quantitative pooling of results was not appropriate and no meta-analysis was performed. Findings were therefore synthesised using a qualitative narrative approach and grouped thematically to summarise prevalence patterns, associated predictors, and prevention strategies. Major heterogeneity sources included variation in haemoglobin measurement techniques, differing anaemia cut-offs, and age-group definitions across studies, which precluded quantitative pooling. While foundational pre-2010 studies were included to provide historical baseline context, greater interpretive emphasis was placed on more recent national surveys and contemporary regional studies when describing current epidemiological patterns.

Protocol Registration

The review protocol was not registered in PROSPERO. Although prospective registration was not undertaken, reporting bias was minimized through the use of predefined eligibility criteria, systematic search strategies, dual-reviewer screening, and adherence to PRISMA guidelines.

Ethical Considerations

Ethical approval was not required as this study involved secondary analysis of published data without direct human participant involvement.

Results

Study Selection

**Figure 1:**
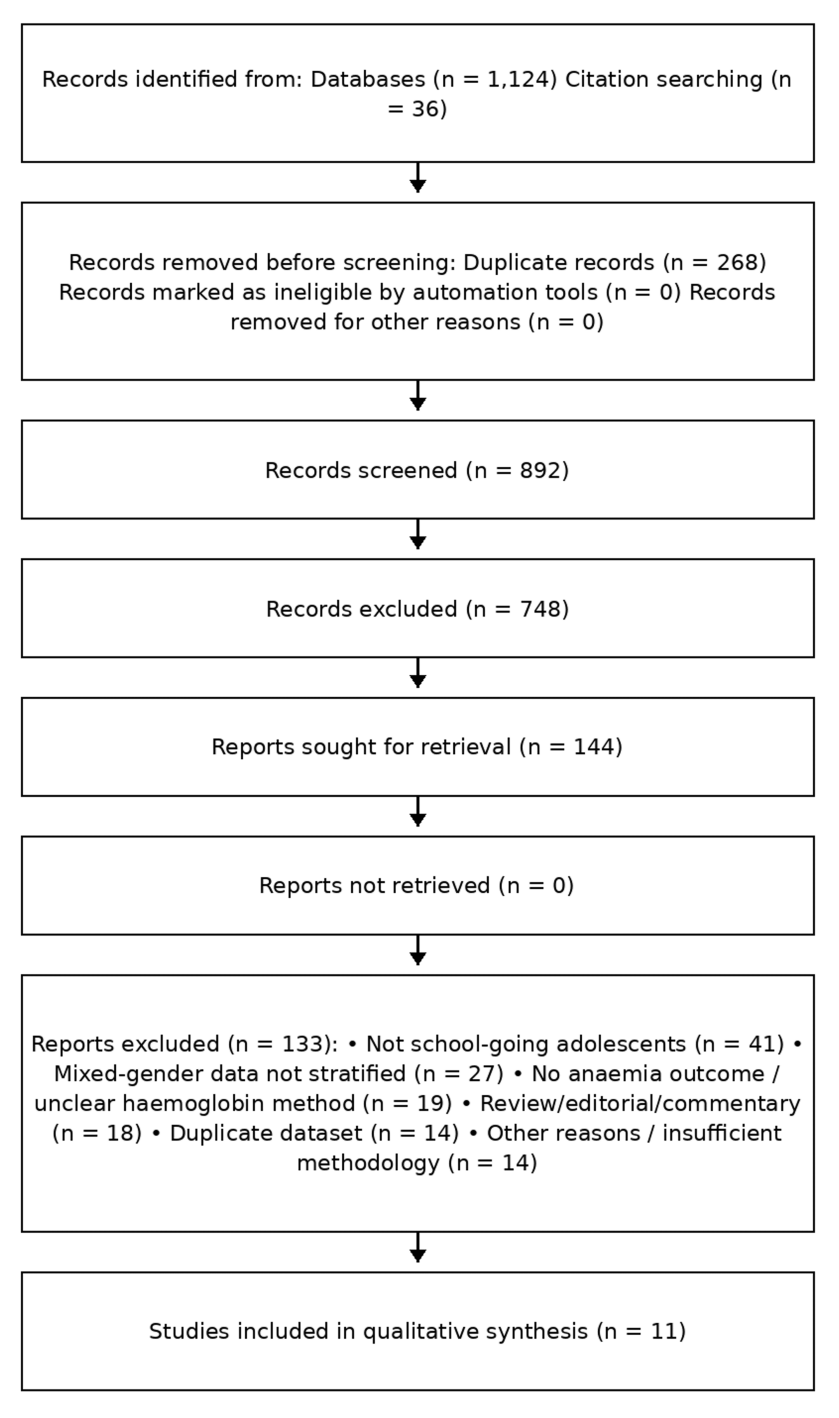
PRISMA 2020 flow diagram of study selection The diagram illustrates the process of identification, screening, eligibility assessment, and inclusion of studies in this systematic review. A total of 1,124 records were identified through database searches and 36 through citation searching. After removal of 268 records prior to screening, 892 records were screened, of which 748 were excluded. A total of 144 full-text articles were assessed for eligibility; 133 were excluded with predefined reasons. Finally, 11 studies were included in the qualitative synthesis. PRISMA: Preferred Reporting Items for Systematic Reviews and Meta-Analyses.

The literature search identified 1,160 records. After removal of records prior to screening, 892 records were screened. Following full-text assessment of 144 articles, 11 studies fulfilled the eligibility criteria and were included in the final synthesis (Figure 1).

Prevalence of Anaemia

National surveys consistently reported high anaemia prevalence among adolescent girls, exceeding 50% in recent estimates [11,12]. Regional school-based studies reported prevalence ranging from approximately 40% to over 70%, with higher burden observed in northern, eastern, and tribal regions [13-21]. This variation likely reflects both geographic differences across regions and methodological heterogeneity, including differences in study design, sampling, and haemoglobin assessment methods. Reporting of anaemia severity categories (mild, moderate, and severe) was not uniform across studies, with several investigations providing only overall prevalence without stratification. Characteristics of studies included in the systematic review (n = 11) are depicted in Table 1.

**Table 1:** Characteristics of studies included in the systematic review (n = 11) Characteristics of studies included in the systematic review of anaemia among school-going adolescent girls in India. CNNS, Comprehensive National Nutrition Survey; NFHS, National Family Health Survey; IIPS, International Institute for Population Sciences; MoHFW, Ministry of Health and Family Welfare; T-3, Test, Treat, and Talk; Hb, haemoglobin.

Author (year)	State/region	Study design	Setting	Sample size (n)	Haemoglobin assessment method	Anaemia prevalence (%)
Sahoo et al. (2023) [21]	Odisha (tribal residential schools)	Cross-sectional	School-based	420	Automated analyser	65.4
Kamble et al. (2021) [18]	Delhi	Cross-sectional	School-based (T-3 camp)	1,020	Automated analyser	56.2
IIPS & ICF (2021) [11]	National	Survey (NFHS-5)	School-going adolescents	≥67,000	Standardised analyser	59.1
Chandrakumari et al. (2019) [19]	Tamil Nadu	Cross-sectional	School-based	430	Automated analyser	54.3
MoHFW (2019) [8]	National	Survey (CNNS)	School-going adolescents	≥20,000	Standardised analyser	40.5
Patil et al. (2018) [17]	Karnataka (North Karnataka school)	Cross-sectional	School-based	1,200	Automated analyser	41.5
Ahankari et al. (2017) [20]	Maharashtra	Cross-sectional	School-based	1,010	Automated analyser	59.3
Sudhagandhi et al. (2011) [16]	Tamil Nadu	Cross-sectional	School-based	270	Automated analyser	71.0
Toteja et al. (2006) [13]	Multi-state (16 districts)	Cross-sectional	School-based	≥13,000	Cyanmethemoglobin method	90.1
Kaur et al. (2006) [14]	Punjab	Cross-sectional	School-based	730	Sahli’s method	59.8
Rajaratnam et al. (2000) [15]	Tamil Nadu	Cross-sectional	School-based	500	Cyanmethemoglobin method	44.8

Predictors of Anaemia

Nutritional deficiencies, particularly low dietary iron intake and poor dietary diversity, were the most frequently reported predictors [15-17]. Biological factors such as menstrual blood loss and rapid growth further increased iron requirements during adolescence [3]. Socioeconomic disadvantage, poor sanitation, and helminth infections were consistently associated with higher anaemia prevalence [18-21]. While some studies employed multivariable analyses to adjust for confounding factors, most reported associations were descriptive in nature, which should be considered when interpreting the identified predictors.

Prevention Pathways

School-based iron-folic acid supplementation, deworming, fortified mid-day meals, and nutrition education were the primary preventive strategies identified [7,8]. Integrated approaches combining supplementation with behaviour change communication demonstrated greater effectiveness than single-component interventions [22-24].

Overall Summary of Findings

Overall, anaemia prevalence among school-going adolescent girls ranged from 40% to >70%, with national surveys indicating prevalence above 50%. Nutritional deficiency, socioeconomic disadvantage, and suboptimal supplementation adherence emerged as the most consistent predictors. School-based integrated interventions demonstrated higher effectiveness than single-component strategies.

Discussion

This systematic review demonstrates that anaemia among school-going adolescent girls in India remains a severe and persistent public health problem, with prevalence consistently exceeding thresholds of public health significance across regions [11-13]. Despite decades of programmatic attention and expansion of supplementation-based strategies, the magnitude of anaemia has declined only marginally, indicating that existing approaches have been insufficient to address its complex and multifactorial determinants in this population [7,8]. For example, national survey data indicate that anaemia prevalence among adolescent girls remains high, with NFHS-5 reporting prevalence exceeding 50%, with only marginal change from earlier survey estimates. These interpretations are based on thematic synthesis of included studies rather than direct comparative analysis and should be considered within the context of the qualitative nature of the review. Variations in haemoglobin assessment methods (e.g., automated analysers, Sahli’s method, cyanmethemoglobin) across studies may have influenced prevalence estimates and limited direct comparability. The methodological quality of the included studies was overall moderate to high, with detailed JBI ratings presented in Table 2.

**Table 2:** Quality appraisal of included studies using the Joanna Briggs Institute (JBI) criteria JBI = Joanna Briggs Institute; High = ≥7 criteria met; Moderate = 4–6 criteria met; Low = ≤3 criteria met. CNNS, Comprehensive National Nutrition Survey; NFHS, National Family Health Survey; IIPS, International Institute for Population Sciences; MoHFW, Ministry of Health and Family Welfare; T-3, Test, Treat, and Talk; Hb, haemoglobin.

Study	Study design	JBI quality rating
Sahoo et al. (2023) [21]	Cross-sectional	Moderate
Kamble et al. (2021) [18]	Cross-sectional	Moderate
IIPS & ICF (2021) [11]	National Survey	High
Chandrakumari et al. (2019) [19]	Cross-sectional	Moderate
MoHFW (2019) [8]	National Survey	High
Patil et al. (2018) [17]	Cross-sectional	Moderate
Ahankari et al. (2017) [20]	Cross-sectional	Moderate
Sudhagandhi et al. (2011) [16]	Cross-sectional	Moderate
Toteja et al. (2006) [13]	Cross-sectional	High
Kaur et al. (2006) [14]	Cross-sectional	Moderate
Rajaratnam et al. (2000) [15]	Cross-sectional	Moderate

Prior reviews have largely focused on women of reproductive age or mixed adolescent populations without isolating school-going cohorts or examining implementation pathways. By explicitly linking epidemiological burden with delivery gaps in school-based platforms, the present review extends earlier adolescent nutrition syntheses that predominantly examined community-based samples or adult women.

Comparison With Existing Evidence

The prevalence ranges identified in this review (approximately 40% to >70%) are comparable to, and in several regions exceed, those reported in earlier national surveys and global burden assessments [4,11,12]. Unlike broader reviews that pool adolescents irrespective of schooling status, this review highlights that school-going adolescent girls continue to experience a burden similar to or higher than community-based estimates, underscoring that school attendance alone does not confer nutritional protection.

Predictors: Beyond Nutritional Deficiency

While iron deficiency remains the predominant proximate cause of anaemia, this review underscores that biological, social, and environmental determinants act synergistically. Menarche-related blood loss and rapid growth spurts substantially increase iron requirements during adolescence [3]. These physiological demands are frequently unmet due to poor dietary diversity and low intake of bioavailable iron [15-17].

Socioeconomic disadvantage, gender-biased intra-household food allocation, household food insecurity, poor sanitation, and helminthic infections were consistently associated with higher anaemia prevalence [18-21]. Several studies indicated that girls often enter anaemia prevention programmes already iron-deficient, suggesting that interventions initiated during adolescence may be insufficient to offset cumulative nutritional deprivation from childhood.

Programmatic Limitations and Implementation Gaps

India’s anaemia control strategies, including the Weekly Iron and Folic Acid Supplementation programme and Anaemia Mukt Bharat, represent some of the most ambitious large-scale nutrition programmes globally [7,8]. However, this review identifies persistent implementation gaps, including poor adherence, inconsistent supply chains, inadequate counselling, and weak outcome monitoring.

Importantly, coverage does not equate to impact. Programmatic and study-based evidence indicate that, despite high programme reach and improved iron-folic acid supplementation coverage, corresponding improvements in haemoglobin outcomes have been limited or inconsistent, highlighting a gap between programme reach and biological outcomes [7,8]. Disruptions to school-based services during the COVID-19 pandemic may have further exacerbated anaemia burden among vulnerable adolescents [22].

School-Based Platforms as a Prevention Opportunity

Schools offer a strategic platform for anaemia prevention through sustained engagement, peer influence, and integration of health education. Evidence suggests that integrated school-based approaches combining supplementation with nutrition education, dietary diversification, menstrual health education, and periodic screening are more effective than single-component interventions [22-25]. Schools function not only as educational institutions but also as community-linked platforms that connect adolescents, families, frontline health workers, and local health systems, positioning them as strategic sites for community-based anaemia prevention.

However, success depends on stronger coordination between health and education sectors, enhanced capacity of frontline workers, and meaningful community engagement.

Policy, Practice, and Research Implications

Implications for research: This review highlights the need to reframe adolescent anaemia control as a life-course and equity issue rather than a standalone supplementation challenge. From a research perspective, longitudinal and implementation studies evaluating scalability, sustainability, and programme fidelity are urgently needed. Although this review focuses on India, the findings may be relevant to other low- and middle-income countries facing similar adolescent nutrition and health-system challenges.

Implications for practice: Anaemia prevention efforts targeting school-going adolescent girls in India should move beyond supplementation coverage alone towards implementation-strengthened, school-based delivery models that integrate nutrition education, menstrual health awareness, dietary diversification, and periodic haemoglobin screening. Community engagement involving parents, teachers, and frontline health workers is essential to improve adherence, acceptability, and long-term sustainability of school-based anaemia prevention programmes.

Implications for policy: National and school health programmes should align iron-folic acid supplementation protocols with World Health Organization recommendations regarding dosage, frequency, and duration to ensure both safety and effectiveness. Policy frameworks should prioritise early preventive interventions beginning in late childhood, strengthen intersectoral coordination between health and education systems, and incorporate robust monitoring mechanisms focused on haemoglobin outcomes rather than distribution metrics alone. Addressing underlying social determinants, such as food insecurity, sanitation, and gender inequities, will be critical to achieving sustainable reductions in adolescent anaemia burden.

Strengths and Limitations

Strengths: Key strengths of this review include its exclusive focus on school-going adolescent girls, use of PRISMA-compliant methodology [9], dual-reviewer screening, and formal methodological quality appraisal using standardised tools [10].

Limitations: Most included studies were cross-sectional in design, limiting causal inference. Considerable heterogeneity was observed in haemoglobin measurement techniques, anaemia cut-off values, and reporting of severity categories, which precluded quantitative meta-analysis. Publication bias cannot be excluded, as grey literature and non-English studies may have been underrepresented. Finally, regional clustering of studies in selected states may limit the national generalisability of the findings. The relatively small number of eligible studies reflects the narrow population definition and limited availability of school-based research in this area, rather than deficiencies in the search strategy. Formal assessment of publication bias (e.g., funnel plot analysis) was not feasible due to the absence of a quantitative meta-analysis.

## Conclusions

This systematic review demonstrates that anaemia among school-going adolescent girls in India remains highly prevalent despite long-standing national nutrition programmes. The burden is driven by a complex interplay of nutritional inadequacy, increased physiological iron requirements during adolescence, socioeconomic disadvantage, poor sanitation, and suboptimal adherence to prevention programmes. While school-based interventions such as iron-folic acid supplementation and deworming are widely implemented, their effectiveness is frequently constrained by implementation gaps and weak outcome monitoring.

Reducing anaemia among school-going adolescent girls will require a shift from coverage-focused strategies towards context-specific, implementation-strengthened interventions embedded within a life-course and equity-oriented framework. Future research should prioritise longitudinal and implementation studies to inform scalable and sustainable anaemia prevention strategies.

## Appendices

**Table 3:** Database search strategies used in the systematic review Note: Search strategies were adapted according to the indexing structure and search interface of each database.

Database	Search strategy
PubMed	(“anaemia” OR “anemia” OR “iron deficiency”) AND (“adolescent girls” OR “school-going adolescents”) AND (“India”) AND (“prevalence” OR “risk factors”)
Scopus	(‘anaemia’ OR ‘anemia’ OR ‘iron deficiency’) AND (‘adolescent girls’ OR ‘school-going adolescents’) AND (‘India’) AND (‘prevalence’ OR ‘risk factors’)
Web of Science	TS=(“anaemia” OR “anemia” OR “iron deficiency”) AND TS=(“adolescent girls” OR “school-going adolescents”) AND TS=(India) AND TS=(“prevalence” OR “risk factors”)
Google Scholar	“anaemia” AND “adolescent girls” AND India AND prevalence
Cochrane Library	anaemia AND “adolescent girls” AND India
